# MicroRNA-92a-3p Enhances Cisplatin Resistance by Regulating Krüppel-Like Factor 4-Mediated Cell Apoptosis and Epithelial-to-Mesenchymal Transition in Cervical Cancer

**DOI:** 10.3389/fphar.2021.783213

**Published:** 2022-01-14

**Authors:** Jing Yang, Jing Hai, Xuecai Dong, Mengjie Zhang, Shufeng Duan

**Affiliations:** Department of Gynecological Oncology I, Xinxiang Central Hospital, The Fourth Clinical College of Xinxiang Medical University, Xinxiang, China

**Keywords:** cervical cancer, cisplatin resistance, KLF4, miR-92a-3p, apoptosis

## Abstract

Recent studies have confirmed the existence and key roles of microRNA (miRNAs) in cancer drug resistance, including cervical cancer (CC). The present study aims to establish a novel role for miR-92a-3p and its associated gene networks in cisplatin (DDP) resistance of CC. First, the disparities in miRNA expression between CC tissues and adjacent normal tissues were screened based on GSE19611 microarray data that retrieved from Gene Expression Omnibus (GEO), and we identified several miRs that were significantly downregulated or upregulated in CC tissues including miR-92a-3p. Moreover, miR-92a-3p was significantly up-regulated in DDP-resistant cells and was the most differently expressed miRNA. Functionally, knockdown of miR-92a-3p increased the sensitivity of DDP-resistant cells to DDP via inhibiting cell proliferation, migration and invasion, and promoting apoptosis. Conversely, overexpression of miR-92a-3p significantly induced DDP resistance in CC parental cells including HeLa and SiHa cells. Moreover, Krüppel-like factor 4 (KLF4) was identified as a direct target of miR-92a-3p, and an obvious inverse correlation was observed between the expression of miR-92a-3p and KLF4 in 40 pairs of cancer tissues. Furthermore, KLF4 knockdown reversed the promoting effect of miR-92a-3p inhibition on DDP sensitivity in DDP-resistant CC cells. Besides, high expression of miR-92a-3p was associated with DDP resistance, as well as a short overall survival in clinic. Taken together, these findings provide important evidence that miR-92a-3p targets KLF4 and is significant in DDP resistance in CC, indicating that miR-92a-3p may be an attractive target to increase DDP sensitivity in clinical CC treatment.

## Introduction

Cervical cancer (CC) is the fourth common cancer among women, which accounts for approximately 7.5% of the total cancer deaths worldwide (Chatterjee et al., 2019). Despite significant improvement in therapy for preventing CC, annually, approximately 500,000 women develop CC worldwide, and about 200,000 die of this disease (Diaz-Padilla et al., 2013). Cisplatin-based concomitant chemoradiotherapy is considered as the standard treatment for patients with advanced/recurrent cervical cancer. However, the clinical usage of cisplatin is limited due to the acquisition of chemotherapy resistance in CC (Amable, 2016). Therefore, studies of the mechanism and reliable prognostic markers to overcome cisplatin resistance are needed to improve outcomes in patients with CC.

Cisplatin (DDP), a platinum-based anticancer agent, is clinically proven to have significant efficacy in different types of cancers including CC (Lorusso et al., 2014). In addition to monotherapy, the anticancer activity of DDP and its analogues has also been demonstrated (Weiss and Christian, 1993). To date, it is estimated that approximately 50% of all patients with cancer will be treated with DDP in their anticancer therapies. Despite major clinical success, prolonged and high-dose DDP always cause serious side effects, which is one of the inherent challenges of this drug to limit its application and effectiveness (Shen et al., 2012). DDP resistance mechanisms are complex and may be related with the reduction of intracellular accumulation drugs; increased DNA repair or inactivation of apoptosis and activation of epithelial-mesenchymal transition (EMT) (Siddik, 2002; Galluzzi et al., 2012; Makovec, 2019). Interestingly, previous studies suggest that DDP resistance can result from epigenetic changes at molecular and cellular levels, including up- or down-regulated expression of microRNA (miRNA) (Croce, 2009).

MicroRNAs (miRNAs) are endogenous small non-coding RNA molecules (19  – 22 bases in length) that are involved in many physiologic and pathologic processes via regulating target gene expression (Bartel, 2009; Hummel et al., 2010; Shukla et al., 2011). During the last decades, increasing evidence has demonstrated that miRNAs play an important role in determining DDP resistance (Cao et al., 2014; Xu et al., 2017), such as miR-574-3p in gastric cancer (Wang et al., 2019), miR-221 in osteosarcoma (Yu WC. et al., 2019), miR-29c-3p in ovarian cancer (Hu et al., 2020). Recently, several studies have demonstrated that miRNAs also have critical role in CC development through modulating tumor growth, invasion, apoptosis, and drug resistance (Di Leva et al., 2012; Santos et al., 2018). For example, in DDP-resistant CC cells, miR-7-5p promoted DDP resistance *via* regulating autophagy and DNA repair activity by targeting BCL2 and PARP-1 genes (Yang et al., 2018). Chen et al. found that miR-499a-5p increased the resistance to DDP both in CC cells and mouse models by targeting SRY-Box Transcription Factor 6 (SOX6) (Chen et al., 2020). Shi et al. showed that miR-144 could overcome the resistance to DDP through targeting LIM homeobox 2 (LHX2) in CC cells (Shi et al., 2019). However, the relationships between miRNAs and DDP resistance remain poorly understood in cervical cancer.

In the present study, we firstly established two DDP-resistant CC cell lines (HeLa/DDP and SiHa/DDP). And then the differentially expressed miRNAs were screened based on GSE19611 microarray data retrieved from GEO, and miR-92a-3p showed the highest change fold. Next, we investigated the roles of miR-92a-3p in cisplatin-resistant cell lines and attempted to reveal the underlying molecular mechanisms. These findings suggest that miR-92a-3p might be utilized in new therapy development for DDP resistance in cervical cancer.

## Methods and Materials

### Tissue Samples

40 pairs of human cervical cancer tissue and adjacent normal tissues samples were obtained from patients who underwent surgical resection at the Department of Gynecological Oncology, Xinxiang Central Hospital, the Fourth Clinical College of Xinxiang Medical University between 2017 and 2018. The samples were snap-frozen in liquid nitrogen and stored at −80°C. The study was approved by the Research Ethics Committee of Xinxiang Medical University. Informed consent was obtained from all patients.

### MicroRNA Expression Profile Data From GEO

MicroRNA data (accession number: GSE19611) from GEO databases in NCBI (http://www.ncbi.nlm.nih.gov/geo/) was downloaded. Differentially expressed miRNAs (DE-miRNAs) between CC tissue samples and adjacent normal tissues were screened based on GEO2R (www.ncbi.nlm.nih.gov/geo/geo2r/), an interactive web tool. The criterion for DE-miRNAs has been set as |fold change| > 1.5 and *p* < 0.01. After normalization, the heat map of expression levels of 58 miRNAs was generated using GeneSpring GX, version 7.3 statistical software.

### RT-qPCR

MiRNA was prepared using the miRNeasy Mini kit (Qiagen, Inc., Germany) according to the manufacturer’s protocol. The concentration and quality of total RNA was determined using TaqMan™ Advanced miRNA Assay (Thermo Fisher Scientific, Inc., Waltham, MA, U.S.A). For the detection of miRNAs expression, 1 μg of total RNA was reversely transcribed into cDNA using Taqman™ microRNA reverse transcription kit (Thermo Fisher Scientific, Inc., Waltham, MA, U.S.A). Taqman miRNA assays (Thermo Fisher Scientific, Inc., Waltham, MA, U.S.A) were used to amplify the expression of miR-92a-3p (Assay ID: 477827_mir), miR-221-3p (Assay ID: 000524_mir), miR-21 (Assay ID: 477975_mir), miR-146a (Assay ID: 478399_mir), miR-196a (Assay ID: 478230_mir), miR-206 (Assay ID: 477968_mir), miR-383 (Assay ID: 478079_mir), miR-497 (Assay ID: 478138_mir) and RNU6B (Assay ID: 001093) in cell lines and tissue samples. PCR reactions were performed in 10-µL reactions using an ABI 7900HT Sequence Detection System with incubations performed at 50°C for 2 min; 95°C for 10 min; and 40 cycles of 95°C for 15 s, 60°C for 1 min. The miRNA primers used for miRNA qPCR assay were all in these kits.

For mRNA analysis, total RNA was extracted from tissues and cells by TRIzol^®^ reagent (Invitrogen; Thermo Fisher Scientific, Inc.). 1 μg total RNA was reverse-transcribed into complementary DNA in the light of PrimeScript RT Kit (Takara Biotechnology Ltd., Liaoning, China) and RT-PCR was conducted using a SYBR Premix Ex Taq II Kit (Applied Biosystems). PCR amplification was performed as follows: 30 thermocycles of 94°C for 30 s, 55°C for 30 s, and 72°C for 30 s on a Bio-Rad CFX96 Real-Time System (Bio-Rad, Hercules, CA, United States). The sequences of the primers used for Krüppel-like factor 4 (KLF4) and GAPDH are as follows: KLF4 forward, 5′-CCC​AAT​TAC​CCA​TCC​TTC​CT-3′ and reverse 5′-AGG​TTT​CTC​ACC​TGT​GTG​GG-3′: GAPDH forward, 5′-CTC​CTC​CTG​TTC​GAC​AGT​CAG​C-3′, and reverse 5′-CCC​AAT​ACG​ACC​AAA​TCC​GTT-3′. Fold changes in the expression of each gene were calculated using the 2^−∆∆Cq^ method (Livak and Schmittgen, 2001).

### Cell Culture and Treatment

Two cervical cancer cell lines (HeLa and SiHa) were purchased from ATCC (Manassas, VA, United States) and cultured in DMEM (Dulbecco’s minimum essential medium) containing 10% fetal bovine serum (FBS, Gibco, Grand Island, NY, United States) at 37°C in a humidified incubator with 5% CO_2_. HeLa and SiHa cells were cultured in increasing concentrations of DDP (Sigma, St. Louis, MO, United States) for over 6 months to establish DDP-resistant cell lines, HeLa/DDP and SiHa/DDP as previously described (Liu et al., 2020). Changes in DDP sensitivity to parent cells and DDP-resistant cells were detected by the Cell Counting Kit-8 (CCK-8) assay (Sigma-Aldrich; Merck KGaA) to determine the successful establishment of drug-resistant cell lines.

Afterwards, the parental cells (HeLa and SiHa) were classified into three groups: mimics NC group, mimics NC + DDP (2 μM) group and miR-92a-3p mimics + DDP (2 μM) group. DDP-resistant cells (HeLa/DDP and SiHa/DDP) were classified into five groups: inhibitor NC group, inhibitor NC + DDP (10 μM) group, miR-92a-3p inhibitor + DDP (10 μM) group, si-scramble group, miR-92a-3p inhibitor + DDP (10 μM) + si-KLF4 group. During transfection, the cells (2×10^5^ cells/well) were plated into 6-well culture plates. The cells were transfected using Lipofectamine™ 2000 (Invitrogen; Thermo Fisher Scientific, Inc.), according to the manufacturer’s protocols. miR-92a-3p mimics, mimics NC, miR-92a-3p inhibitor, inhibitor NC, KLF4 siRNA and their negative control (NC) were purchased from Genephama Biotech (Shanghai, China). After 48 h, cells were selected for subsequent experiments.

### Cell Viability Assay

Cells were seeded in the 96-well plates (0.5 × 10^4^ cells per well) and cultured until they reached 80% confluence, and the aforementioned miRs and negative controls were transfected into the parent cells and DDP-resistant cells. After 24 h transfection, DDP (at different concentrations) was added into above cells. After 48 h incubation, cell viability was assessed by the CCK-8 assay. Briefly, 10 μL CCK-8 solutions were added to each well at 4 h before the endpoint of incubation. OD_450nm_ value in each well was determined by a microplate reader (BioTek, Winooski, VT, United States). The median inhibition concentration of each drug (IC_50_) was estimated by the relative survival curve.

### Caspase-3 Activity Assay

A total of 5 × 10^4^ cells were plated in a 10-cm Petri dish for a 24-h growth period. After 48 h of DDP treatment, as indicated above, cells were harvested and caspase-3 activity was determined using the Caspase-3 assay kit (cat. no. C1115, Beyotime Institute of Biotechnology, Shanghai, China). Caspase-3 activity assay was performed on 96-well plates by incubating 10 μL protein of cell lysate per sample in 80 μL reaction buffer containing 10 μL caspase-3 substrate (Ac-DEVD-pNA; 2 mM) at 37°C for 2 h according to the manufacturer’s protocol. The optical density at 405 nm was then detected using a microplate reader (Model 680; Bio-Rad Laboratories, Inc.).

### Apoptosis Assay

After 48 h of DDP treatment, as indicated above, the percentage of apoptotic cells was detected using An annexin V-FITC/propidium iodide (PI) apoptosis detection kit (Beyotime, China) according to the manufacturer’s guidelines. After the cells were harvested and resuspended in PBS, 10 μL of ready-to-use annexin V-FITC (Beyotime Institute of Biotechnology, Shanghai, China) was added into the mixture. The cells were incubated at 37°C for 15 min and counterstained with 5 μL of PI in the dark for 30 min. Then the percentages of apoptotic cells from each group were examined by using BD FACSDiva 6.1.3 software (BD Biosciences, United States), and the results were analyzed using CellQuest software (BD Bioscience). Annexin V-positive cells were regarded as apoptotic cells.

### Cell Invasion Assays

Transwell membranes with 8 μm pores (Coring, NY, United States) were used to evaluate cell invasion. Briefly, after 48 h of DDP treatment, as indicated above, 1.0×10^5^ cells were seeded on the top chamber pre-coated with Matrigel (Coring, NY, United States). The matched bottom chambers were filled with 500 μl 10% FBS in DMEM. After 48 h incubation at 37°C, non-invaded cells on the upper surface were removed using a cotton swab and invaded cells on the lower membrane surface were fixed in cold methanol for 20 min at room temperature, and then stained with 0.1% crystal violet, photographed at 100 × magnification under a microscope (Olympus IX81, Tokyo, Japan) and the migrated cells were counted by averaging the total number of cells from triplicate determinations.

### Wound Healing Assay

After 48 h of DDP treatment, as indicated above, the cell monolayer was scraped using the tip of 10 μl pipette, and the debris was rinsed with phosphate-buffered saline (PBS). Serial photographs were obtained at 0 and 48 h using a phase contrast microscope at 100 × magnification (Olympus IX81, Tokyo, Japan). The wound healing rate was calculated by using the ImageJ software (version 1.46; National Institutes of Health, Bethesda, MD, United States).

### Immunofluorescence Assay

Cells were seeded into a 24-well cell culture plate at a density of 2×10^5^ cells/well. When cell confluence reached 80% ∼90%, the aforementioned miRs and negative controls were transfected into the parent cells and DDP-resistant cells. After 24 h transfection, DDP (at different concentrations) was added. After 48 h, the cells were fixed with 4% paraformaldehyde and blocked with 5% FBS containing 0.5% Triton X-100 for 5  min. Subsequently, the cells were incubated with cleaved-caspase 3 (cat. no. ab208003, 1:1,000 dilution, Abcam, Cambridge, MA, United States) in phosphate buffer saline (PBS) for 1 h at 37°C. After being washed three times with PBS, the cells were incubated with diluted Fluor 488-conjugated anti-rabbit antibody (cat. no. ab150081, 1:200 dilution, Abcam, Cambridge, MA, United States) for 1 h at 37°C. After each of the above steps, cells were washed by PBS three times.Images were attained under fluorescence microscope.

### Western Blot

Total cellular proteins were lysed in RIPA lysis buffer (Santa Cruz Biotechnology, Inc.). The proteins (25 µg/line) were separated by 10% SDS-PAGE gels and transferred to polyvinylidene difluoride (PVDF) membranes (GE Healthcare; Cytiva). After blocking with 5% skim milk solution for 2  h at room temperature, the membranes were incubated with specific primary antibodies including KLF4 (cat. no.12173, 1:1,000 dilution), E-cadherin (cat. no. 3195, 1:1,000 dilution), N-cadherin (cat. no. 13116, 1:1,000 dilution), Vimentin (cat. no. 5741, 1:1,000 dilution) and *β*-actin (cat no.4970, 1:1000) at 4°C overnight. All above antibodies were obtained from Cell Signaling Technology. Subsequently, the mouse anti-rabbit IgG-HRP secondary antibody (cat no. sc2537, Santa Cruz Biotechnology, Inc., 1:1,000 dilution) was added to the membranes followed by incubation for 2 h at room temperature. The proteins were visualized with an ECL kit (Thermo Fisher Scientific, Inc.). Semi-quantification was performed using ImageJ software (version 1.46; National Institutes of Health, Bethesda, MD, United States).

### Luciferase Assays

The luciferase report plasmids used in this study were constructed by inserting the common KLF4 3′-UTR into the XbaI restriction site located downstream of the luciferase reporter gene in the pGL3-Promoter vector. The sequence from +2486 to +2508 in the human KLF4 mRNA (5′-TGA​ATT​GTG​TAT​TGA​TGC​AAT​AT-3′) was termed the miR-92a-3p recognition element. Subsequently, primers (sense, 5′-CCG *TCT​AGA* GTG​ACT​GGA​AGT​TGT​GGA​TAT​C-3′; and antisense, 5′-CCG *TCT​AGA* CCT​CTT​CTT​CTA​ACA​TCA​T-3′) modified with the XbaI digestion site at both ends were used to amplify the KLF4 3′-UTR (corresponding to nucleotides +2414 to +2577 of the human KLF4 mRNA (NCBI Reference Sequence: NM_001314052.2), and 165 nucleotides in length). The PCR-amplified region was cloned into the XbaI restriction site of the pGL3-promoter vector (Promega, Madison, WI, United States) using standard protocols. The mutated KLF4 3′UTR was commercially synthesized by Tsingke Biological Technology (Beijing, China) and inserted into pGL3 luciferase reporter vector in the same way. In mutated KLF4 3′UTR, the original miR-92a-3p binding sites TGCAATA were changed as CATGCAG. Nucleotide sequences of all plasmids used in this study were confirmed by DNA sequencing analysis. When cells reached 70–80% fusion degree, 40 ng of wt (mut)-KLF4-PGL3 and 40 nM miR-control, miR-92a-3p mimics were co-transfected into HeLa and SiHa. After 48 h incubation, the luciferase activities were measured using the Dual-Luciferase Reporter Assay System (Promega, Madison, WI, United States), according to the manufacturer’s protocol.

### Statistical Data Analysis

Statistical significance was tested using SPSS software (Version 16.0; SPSS Inc.). Data are reported as mean ± SD. Unpaired Student’s t test was used to perform comparison of parameters between the two groups. Using analysis of variance (ANOVA) multiple group comparisons were realized and obeyed Tukey’s post hoc test. Survival rates were calculated using the Kaplan-Meier method and comparisons were performed using the Log-rank test. The correlation between the expression of miRNA and KLF4 was analyzed using Pearson’s correlation analysis. The *p*-value of less than 0.05 was considered statistically significant.

## Results

### Mir-92a-3p Was Up-Regulated in DDP-Resistant CC Cells

**FIGURE 1:**
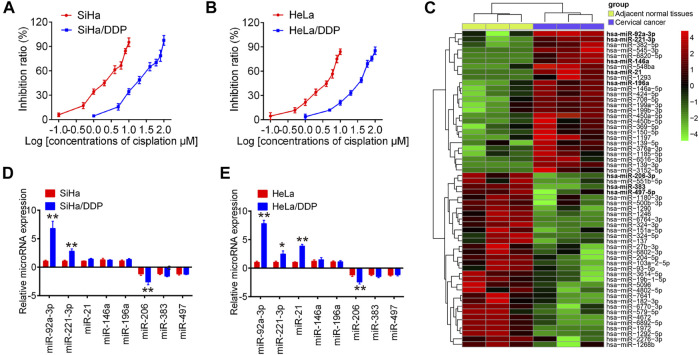
The expression of miRNAs in cisplatin-sensitive and -resistant cervical cancer cells. Effects of cisplatin at different concentrations on cell viability were analyzed using CCK-8 assay in SiHa and SiHa/DDP **(A)**, and in HeLa and HeLa/DDP **(B)**. **(C)** Differentially expressed miRNAs were analyzed between cervical cancer tissues and the adjacent normal tissues group. Data were retrieved from Gene Expression Omnibus (GEO) dataset, with the accession number GSE19611. The color code in the heat map is linear and the expression levels of miRNAs that were upregulated are shown in green to red, whereas the miRNAs that were downregulated are shown from red to green. **(D, E)** miR-92a-3p, miR-221-3p, miR-21, miR-146a, miR-196a, miR-383, miR-206 and miR-497 were further analyzed using qRT-PCR in SiHa/DDP and HeLa/DDP cells. Data are presented as means ± SD of three individual experiments. *p < 0.05, **p < 0.01 *vs*. SiHa or HeLa cells.

First, we developed two DDP-resistant CC cell lines, HeLa/DDP and SiHa/DDP, by treating HeLa and SiHa with escalating DDP concentrations over 6  months. Compared to parental cells, these resistant cells responded poorly to DDP, with almost 10 times higher IC_50_ values than parental cells, where the IC_50_ of SiHa and HeLa for DDP was 2.19 and 3.73 µM, while the IC_50_ of SiHa/DDP and HeLa/DDP was 20.78 and 33.34 µM, respectively (Figures 1A,B). To screen several miRNAs associated with DDP resistance in cervical cancer, we generated miRNA profiles by retrieving microRNA data (accession number: GSE19611) from GEO databases. Cluster analysis indicated a significant difference between cervical cancer tissues and the adjacent normal tissues (Figure 1C). Subsequently, the expression levels of eight differentially expressed miRNAs that have been previously identified in cervical cancer were detected in resistant cells by RT-qPCR (Hou et al., 2014; Hu et al., 2018; Hu et al., 2019; Li et al., 2018; Tao et al., 2018; Wang and Tian, 2018; Wei et al., 2017; Zhang G. et al., 2018). As shown in Figures 1D,E, two of which (miR-92a-3p and miR-221-3p) were significantly increased, one of which (miR-206) were remarkably decreased in both SiHa/DDP and HeLa/DDP cells compared to respective parental cells. Importantly, miR-92a-3p expression levels were the most abundant among these miRNAs. Additionally, miR-92a-3p has been reportedly associated with cancer chemoresistance in breast cancer and gastric cancer (Cun and Yang, 2018; Tao et al., 2019); therefore, miR-92a-3p was chose for further research.

### Knockdown of miR-92a-3p Re-sensitized DDP Resistance Cervical Cancer Cells to DDP

**FIGURE 2:**
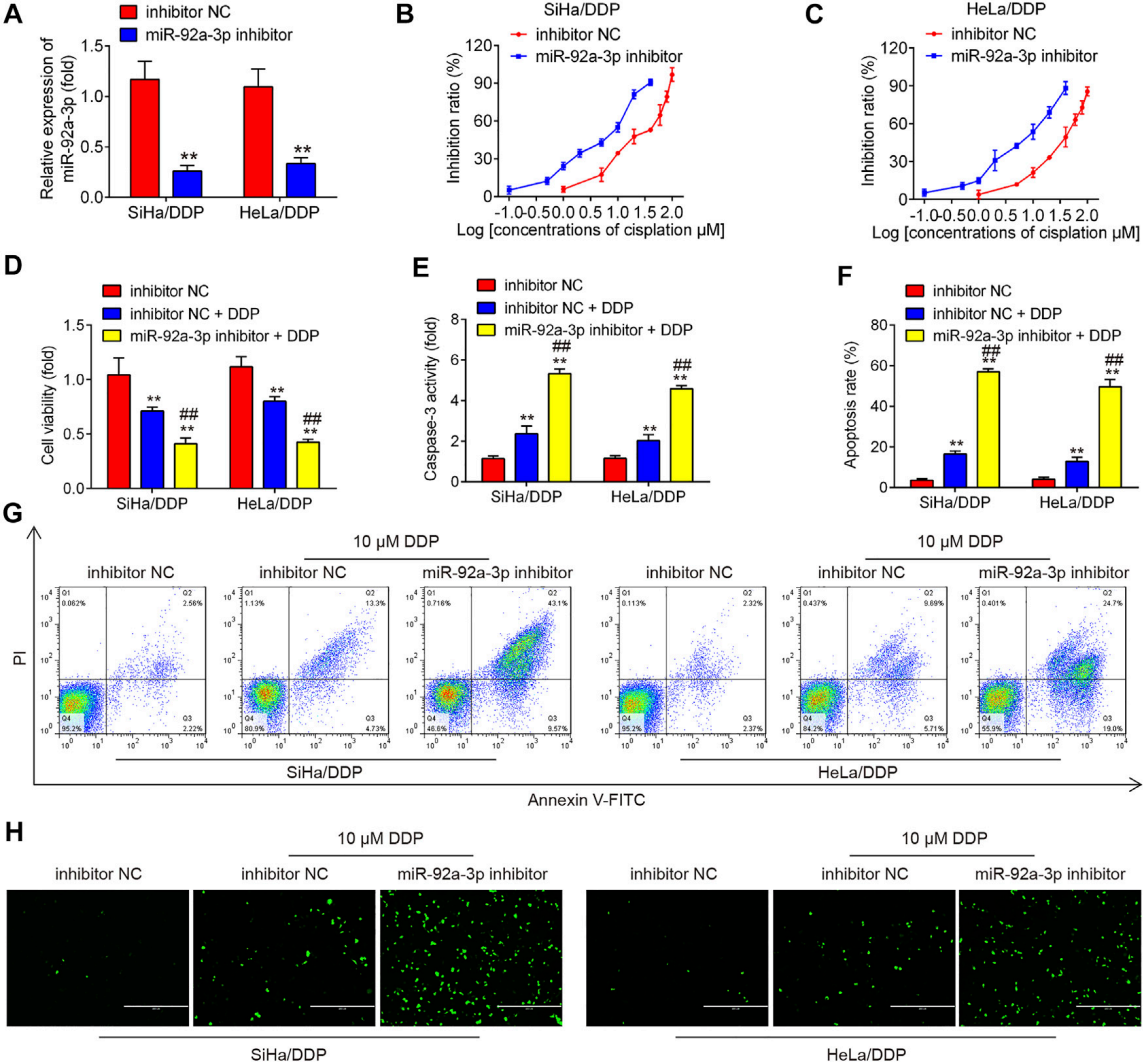
Knockdown of miR-92a-3p increased the sensitivity of DDP resistant cells to DDP. DDP resistant cells (HeLa/DDP and SiHa/DDP) were treated with miR-92a-3p inhibitor or inhibitor NC for 24 h, followed by treatment with 10 μM DDP for 48 h. **(A)** miR-92a-3p expression was detected by qRT-PCR. **(B–D)** Effects of 10 μM DDP on cell viability were analyzed using CCK-8 assay in miR-92a-3p inhibitor transfected SiHa/DDP and HeLa/DDP cells. **(E)** The activity of caspase 3 was measured by a Caspase-3 Activity kit. **(F, G)** The apoptosis rates were analyzed by Flow Cytometry in HeLa/DDP and SiHa/DDP cells. **(H)** The protein levels of cleaved caspase 3 was detected by IFA. Data are presented as means ± SD of three individual experiments. **p < 0.01 *vs*. Inhibitor NC group; ##*p* < 0.01 *vs*. Inhibitor NC + DDP group.

To further elucidate the role of miR-92a-3p in DDP resistance, DDP resistant cells (HeLa/DDP and SiHa/DDP cells) were treated with miR-92a-3p inhibitor. As expected, resistant cells transfected with miR-92a-3p inhibitor showed miR-92a-3p levels were significantly lower than inhibitor negative control (NC) transfected cells (Figure 2A). Inhibition of miR-92a-3p significantly sensitized SiHa/DDP cells to DDP, with the IC_50_ value decreased from 20.78 to 5.27 µM (Figure 2B). Similar results were found in HeLa/DDP cells, where the IC_50_ for DDP decreased from 33.34 to 6.85 µM (Figure 2C). In inhibitor NC transfected HeLa/DDP and SiHa/DDP cells, a slight decrease exhibited in proliferation, while a slight increase was presented in caspase 3 activity and apoptosis rate after 10 µM DDP treatment (Figures 2D–G). Moreover, inhibition of miR-92a-3p enhanced the inhibitory effects of DDP on cell viability (Figure 2D). The caspase 3 activity assay revealed that inhibition of miR-92a-3p enhanced the effects of DDP with promotion of caspase 3 activity in resistant cells (Figure 2E). Flow cytometric analysis showed that inhibition of miR-92a-3p increased cell apoptosis following DDP treatment in resistant cells (Figures 2F,G). In addition, we also measured the protein levels of cleaved caspase 3 by IFA in SiHa/DDP and Hela/DDP cells. As shown in Figure 2H, there is a slight increase in protein levels of cleaved caspase 3 after 10 µM DDP treatment, while inhibition of miR-92a-3p significantly enhanced the protein levels of cleaved caspase 3 in SiHa/DDP and Hela/DDP cells under 10 µM DDP stimulation. Collectively, all these data suggest that decreased miR-92a-3p expression re-sensitized DDP resistance cervical cancer cells to DDP.

### Overexpression of miR-92a-3p Induced DDP Resistance of CC Parental Cells

**FIGURE 3:**
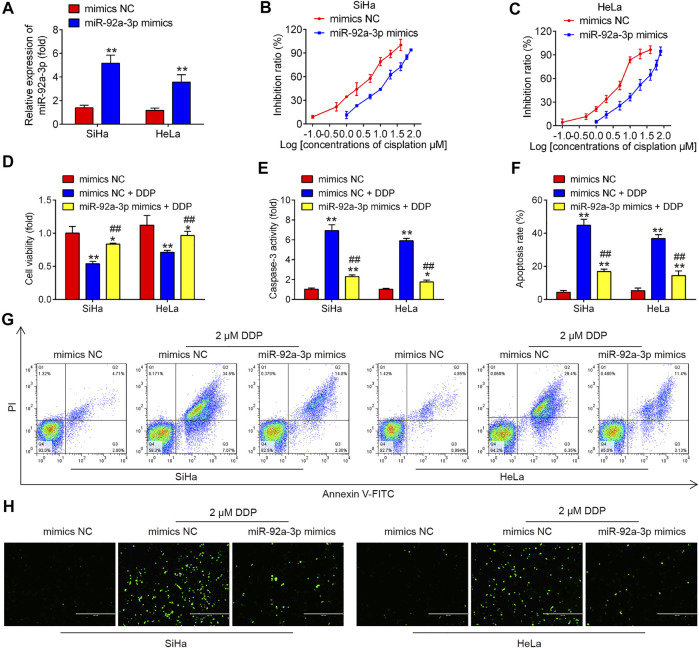
Overexpression of miR-92a-3p induced DDP resistance of cervical cancer cells. Parental HeLa and SiHa cells were treated with miR-92a-3p mimics or mimics NC for 24 h, followed by the treatment with 2 μM DDP for 48 h. **(A)** miR-92a-3p expression was detected by qRT-PCR. **(B–D)** Effects of 2 μM DDP on cell viability was analyzed using CCK-8 assay in miR-92a-3p mimics transfected SiHa and HeLa cells. **(E)** The activity of caspase 3 was measured by a Caspase-3 Activity kit. **(F, G)** The apoptosis rates were analyzed by Flow Cytometry. **(H)** The protein levels of cleaved caspase 3 was detected by IFA. Data are presented as means ± SD of three individual experiments. *p < 0.05, **p < 0.01 *vs*. mimics NC group; ##*p* < 0.01 *vs*. mimics NC + DDP group.

Next, we overexpressed miR-92a-3p in parental HeLa and SiHa cells by transfection of miR-92a-3p mimics. As expected, miR-92a-3p expression levels were notably increased in SiHa and HeLa cells after miR-92a-3p mimics transfection (Figure 3A). After 24 h transfection, we treated the cells with DDP for 48  h at a concentration of 2 μM and found that overexpression of miR-92a-3p decreased the DDP-induced cell cytotoxicity compared to that of mimics NC-transfected cells, with the IC_50_ value increased from 2.19 to 10.61 µM in SiHa cells (Figure 3B). Similar results were also found in HeLa cells, where the IC_50_ for DDP increased from 3.73 to 16.74 µM (Figure 3C). In mimics NC transfected HeLa and SiHa cells, a significant increase exhibited in proliferation, while a marked decrease was presented in caspase 3 activity and apoptosis rate after 2 μM DDP treatment (Figures 3D–G). Moreover, overexpression of miR-92a-3p significantly reversed DDP-induced inhibition of cell viability in parental HeLa and SiHa cells (Figure 3D). The caspase 3 activity assay revealed that overexpression of miR-92a-3p attenuated the effects of DDP with promotion of caspase 3 activity in SiHa and HeLa cells (Figure 3E). Flow cytometric analysis showed that overexpression of miR-92a-3p significantly decreased cell apoptosis following DDP treatment in SiHa and HeLa cells (Figures 3F,G). In addition, IFA assay showed that there is a significant increase in protein levels of cleaved caspase 3 in SiHa and HeLa cells after 2 μM DDP treatment, while overexpression of miR-92a-3p significantly attenuated the promoting effect of DDP on protein levels of cleaved caspase 3 (Figure 3H). Collectively, all these data suggest that overexpression of miR-92a-3p reduced the sensitivity of CC cells to DDP.

### Knockdown of miR-92a-3p Enhanced the Inhibitory Effects of DDP on the Migratory and Invasive Abilities of DDP-Resistant CC Cells

**FIGURE 4:**
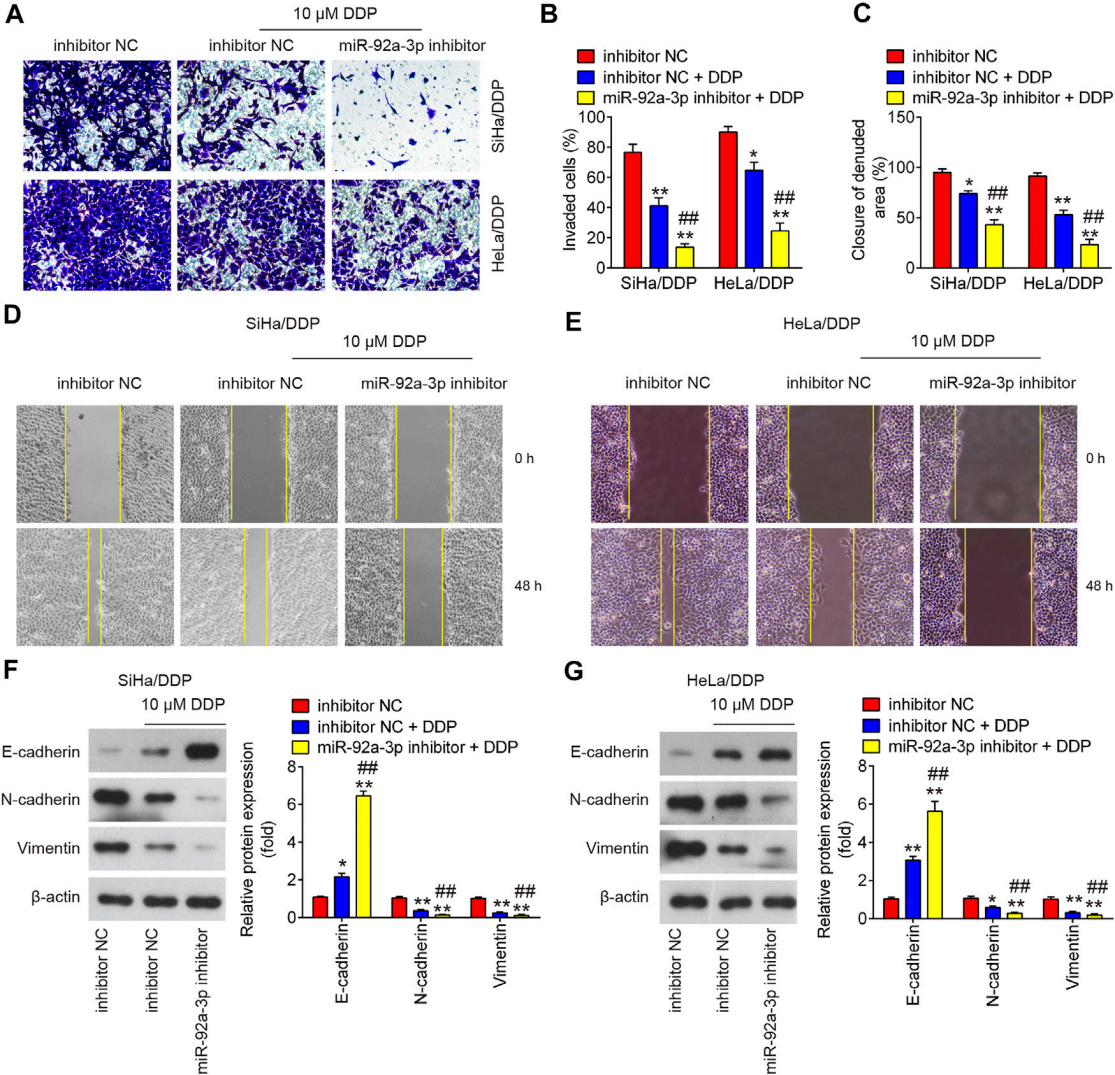
The effects of DDP on the migration, invasion and EMT process in miR-92a-3p inhibitor transfected DDP-resistant cervical cancer cells. DDP resistant cells (HeLa/DDP and SiHa/DDP) were treated with miR-92a-3p inhibitor or inhibitor NC for 24 h, followed by treatment with 10 μM DDP for 48 h. **(A, B)** Effects of 10 μM DDP on cell invasion was analyzed using Transwell assay in miR-92a-3p inhibitor transfected SiHa/DDP and HeLa/DDP cells. **(C, D, E)** Effects of 10 μM DDP on the migration was detected by wound healing assay in miR-92a-3p inhibitor transfected HeLa/DDP and SiHa/DDP cells. **(F, G)** The epithelial protein (E-cadherin) and mesenchymal proteins (Vimentin and N-cadherin) were measured by western blot assay. Data are presented as means ± SD of three individual experiments. **p < 0.01 *vs*. Inhibitor NC group; ##*p* < 0.01 *vs*. Inhibitor NC + DDP group.

To investigate the effect of miR-92a-3p on the migratory and invasive abilities of DDP-resistant CC cells, the matrigel-transwell and wound healing assays were performed. As shown in Figures 4A–E, 10 µM DDP treatment showed slight effect on DDP-resistant CC cells invasion and migration, whereas miR-92a-3p knockdown significantly enhanced the inhibitory effects of DDP on the invasion and migration of SiHa/DDP and HeLa/DDP cells. As epithelial-mesenchymal transition (EMT) is one of the key events in tumor cell chemoresistance (Miow et al., 2015), we examined the EMT related protein using western blot. It was shown that 10 μM DDP treatment slightly upregulated the epithelial protein (E-cadherin), whereas slightly downregulated mesenchymal proteins (Vimentin and N-cadherin) in DDP-resistant cells when compared to inhibitor NC transfected cells; however, these effects of DDP were enhanced by miR-92a-3p knockdown in SiHa/DDP and HeLa/DDP cells (Figures 4F,G). This suggests that decreased miR-92a-3p expression could enhanced the inhibitory effect of DDP on EMT progress.

### Overexpression of miR-92a-3p Resisted the Inhibitory Effects of DDP on Migration, Invasion and EMT Process of CC Parental Cells

**FIGURE 5:**
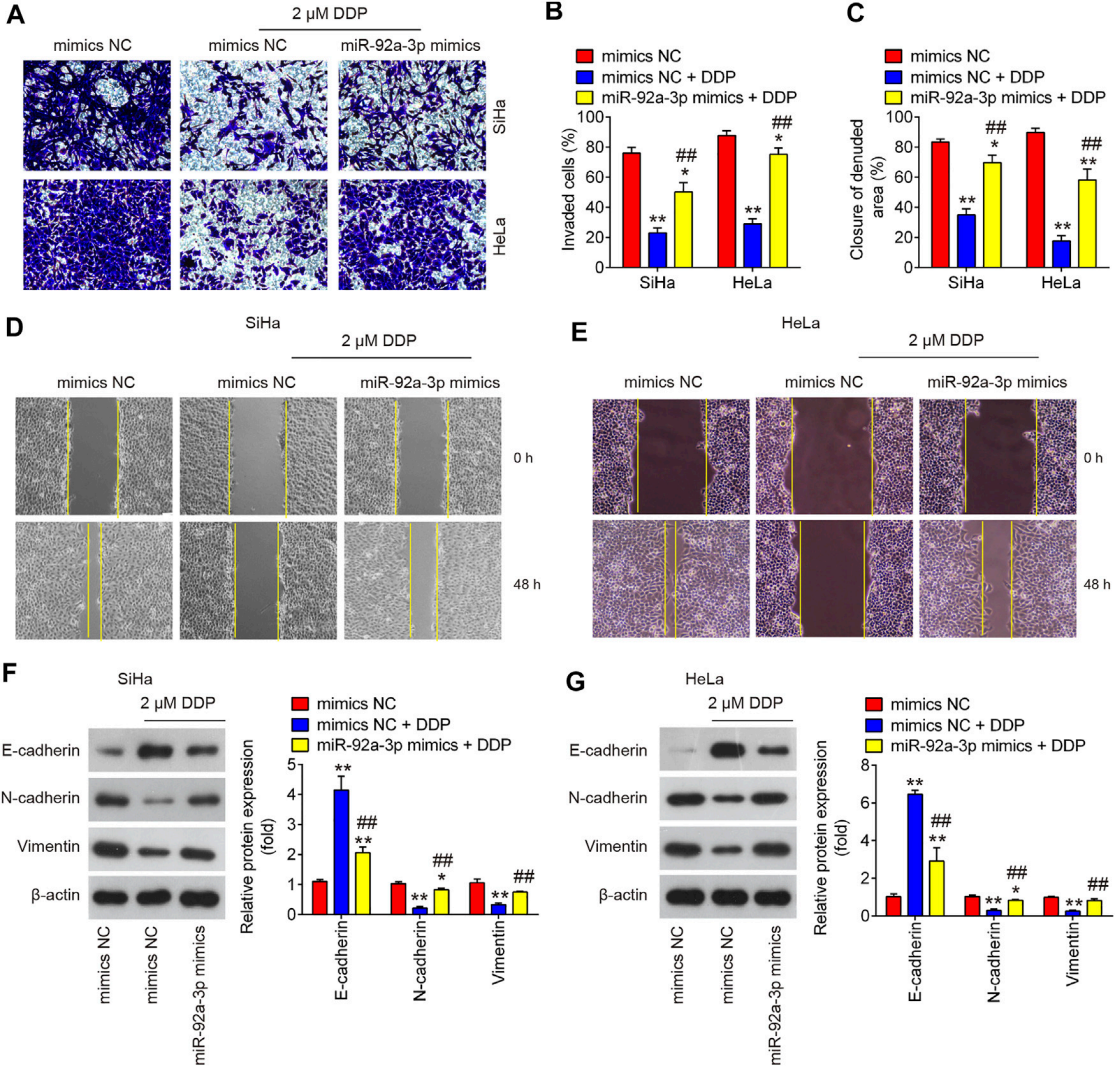
Overexpression of miR-92a-3p promoted DDP resistance in CC parental cells. Parental HeLa and SiHa cells were treated with miR-92a-3p mimics or mimics NC for 24 h, followed by the treatment with 2 μM DDP for 48 h. **(A, B)** Effects of 2 μM DDP on cell invasion was analyzed using Transwell assay in miR-92a-3p mimics transfected SiHa and HeLa cells. **(C, D, E)** Effects of 2 μM DDP on the migration was detected by wound healing assay in miR-92a-3p mimics transfected HeLa and SiHa cells. **(F, G)** The epithelial protein (E-cadherin) and mesenchymal proteins (Vimentin and N-cadherin) were measured by western blot assay. Data are presented as means ± SD of three individual experiments. **p < 0.01 *vs*. mimics NC group; ##*p* < 0.01 *vs*. mimics NC + DDP group.

To further explore the roles of miR-92 in DDP-resistance, we also overexpressed miR-92a-3p in parental HeLa and SiHa cells by transfection of miR-92a-3p mimics. After 24 h transfection, the cells were treated with DDP for 48 h at a concentration of 2 μM and found that DDP treatment significantly inhibited the invasive and migratory abilities of CC cells compared with mimics NC transfected cells; however, these inhibitory effects of DDP were attenuated in miR-92a-3p mimics transfected HeLa and SiHa cells (Figures 5A–E). Western blot analysis showed that low concentration of DDP (2 μM) significantly increased E-cadherin expression and decreased Vimentin and N-cadherin expressions, however, the inhibitory effect of DDP on EMT process was markedly reduced after miR-92a-3p overexpression in SiHa and HeLa cells (Figures 5F,G). Collectively, all these data suggest that overexpression of miR-92a-3p increased DDP resistance of CC parental cells by promoting migration, invasion and EMT.

### KLF4 Is a Direct Target of miR-92a-3p

**FIGURE 6:**
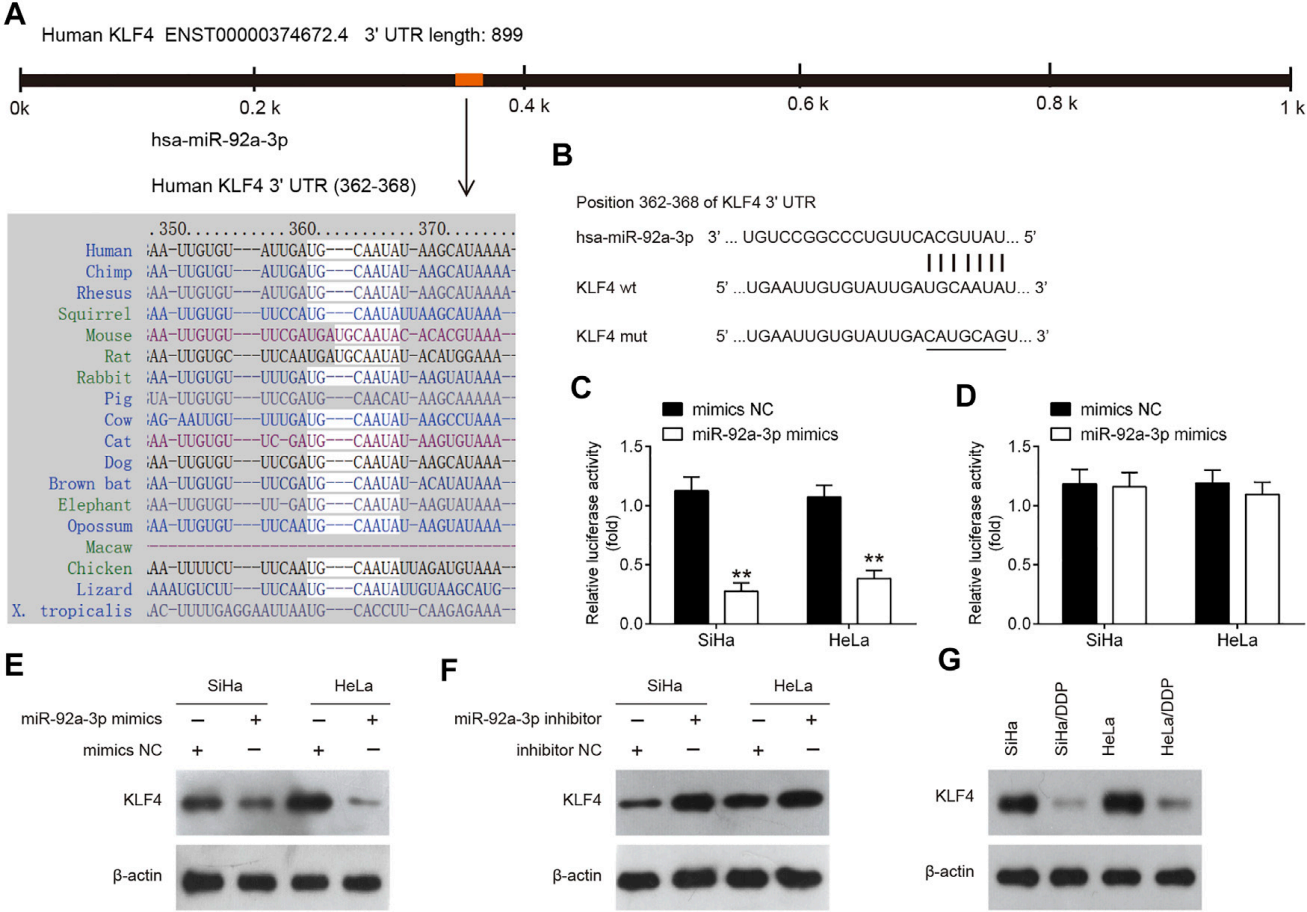
KLF4 was a direct target of miR-92a-3p. **(A)** miR-92a-3p sequence is shown to be highly conserved among species. **(B)** The predicted complementary sequences for miR-92a-3p in the 3′UTR of KLF4 and the mutations are shown in the seed region of miR-92a-3p. **(C, D)** The SiHa and HeLa cells were co-transfected with either pGLO-KLF4-3ʹ-UTR or pGLO-KLF4-mut-3ʹ-UTR, and miR-92a-3p mimics or corresponding NC and the relative luciferase activity were measured. **p < 0.01 *vs* mimics NC. **(E, F)** The SiHa and HeLa cells were transfected with miR-92a-3p mimics/inhibitor or corresponding NC, and the KLF4 protein level was measured using western blot analysis. **(G)** The KLF4 protein level was measured in SiHa/DDP and HeLa/DDP cells.

To identify the mediators of miR-92a-3p-driven DDP resistance, TargetScan and Miranda were performed. This bioinformatic analysis revealed the 3′-UTR of KLF4 contain a predicted binding site for miR-92a-3p (Figures 6A,B). To verify whether KLF4 is a direct target of miR-92a-3p, dual-Luciferase reporter system with pGL3 reporter plasmid containing wild-type or mutant 3′-UTR of KLF4 was used. As shown in Figures 6C,D, co-transfection of miR-92a-3p mimics significantly suppressed the luciferase activity of the reporter containing wild-type 3′-UTR, but not the mutant reporter. Western blot assay indicated that KLF4 protein levels were significantly decreased by miR-92a-3p overexpression, while increased by miR-92a-3p knockdown (Figures 6E,F). These data reveal that KLF4 directly targeted by miR-92a-3p. In addition, we also detected the levels of KLF4 in DDP-resistant CC cells. As shown in Figure 6G, compared with parental HeLa and SiHa cells, KLF4 expression was significantly decreased in SiHa/DDP and HeLa/DDP cells, which indicated that miR-92a-3p maybe involved in DDP-resistance by targeting KLF4.

### KLF4 Is Responsible for miR-92a-3p-Mediated DDP Resistance

**FIGURE 7:**
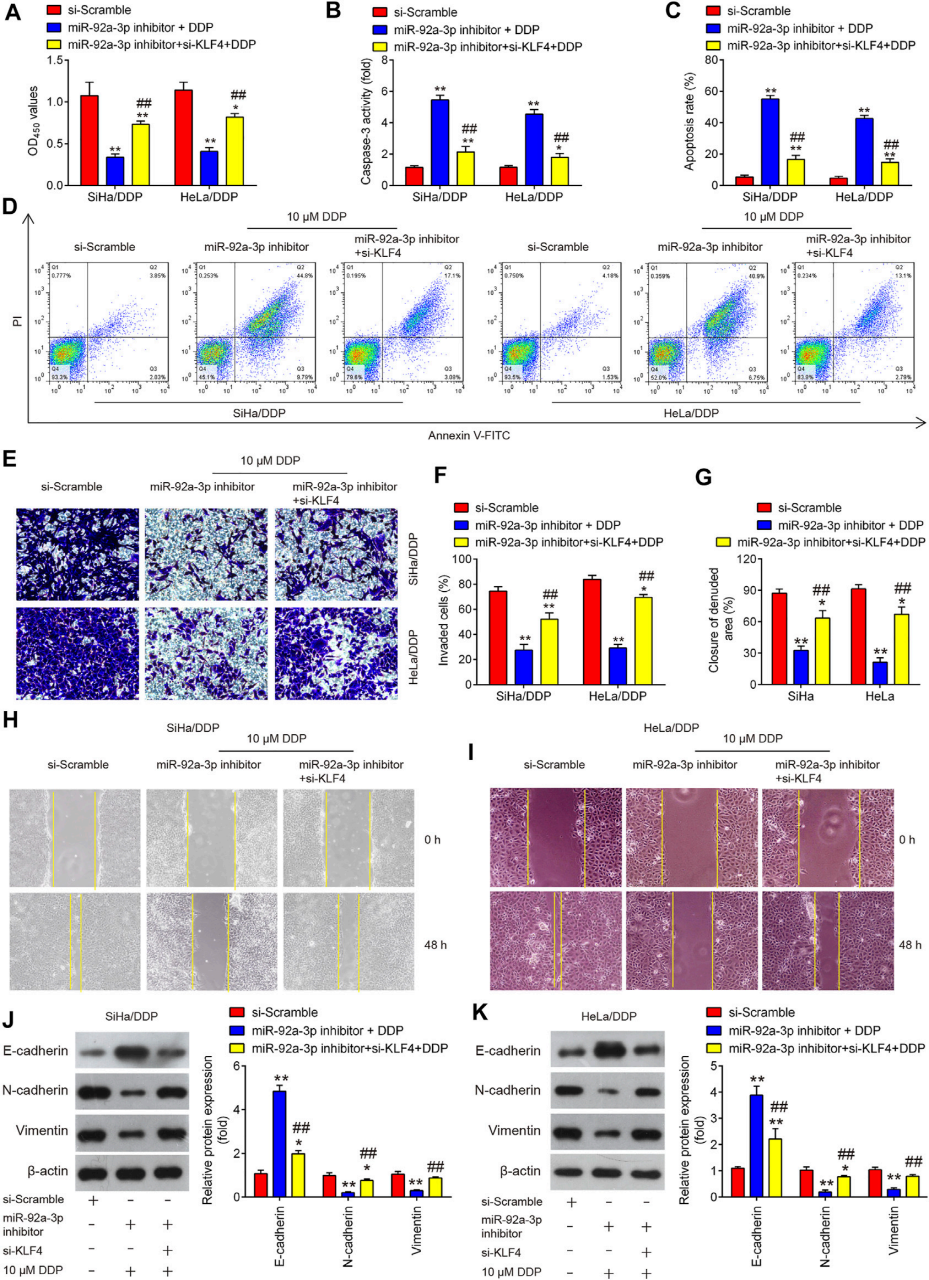
KLF4 is responsible for miR-92a-3p-mediated DDP resistance. HeLa/DDP and SiHa/DDP cells were co-transfected with miR-92a-3p inhibitor and si-KLF4, followed by the treatment with 10 μM DDP for 48 h. **(A)** Effects of 10 μM DDP on cell viability was analyzed using CCK-8 assay in miR-92a-3p inhibitor and si-KLF4 cotransfected SiHa/DDP and HeLa/DDP cells. **(B)** The activity of caspase 3 was measured by a Caspase-3 Activity kit. **(C, D)** The apoptosis rates were analyzed by Flow Cytometry. **(E, F)** Effects of 10 μM DDP on cell invasion was analyzed using Transwell assay in miR-92a-3p inhibitor and si-KLF4 cotransfected SiHa/DDP and HeLa/DDP cells. **(G–I)** Effects of 10 μM DDP on the migration was detected by wound healing assay in miR-92a-3p inhibitor and si-KLF4 cotransfected SiHa/DDP and HeLa/DDP cells. **(J, K)** E-cadherin, Vimentin and N-cadherin protein expression levels were measured by western blot assay in miR-92a-3p inhibitor and si-KLF4 cotransfected SiHa/DDP and HeLa/DDP cells. Data are presented as means ± SD of three individual experiments. *p < 0.05, **p < 0.01 *vs*. si-Scramble group; ##*p* < 0.01 *vs*. miR-92a-3p inhibitor + DDP group.

To explore whether KLF4 involved in the miR-92a-3p-mediated DDP resistance, HeLa/DDP and SiHa/DDP cells were co-transfected with miR-92a-3p and si-KLF4 for 24 h, followed by 10 μM DDP treatment. Subsequently, the viability, apoptosis, invasion and migration of DDP-resistant cells were investigated. We found miR-92a-3p knockdown increased the sensitivity of HeLa/DDP and SiHa/DDP cells to DDP, as evidenced by the reduction of cell viability (Figure 7A), promotion of caspase 3 activity (Figure 7B) and apoptosis (Figures 7C,D). In contrast, when si-KLF4 and miR-92a-3p inhibitor were co-transfected into DDP-resistant cells, the increased sensitivity of miR-92a-3p inhibitor-transfected HeLa/DDP and SiHa/DDP cells to DDP was remarkably reduced, as reflected by the induction of cell viability, reduction of caspase 3 activity and apoptosis. As expected, the miR-92a-3p knockdown markedly decreased the cell invasion and inhibited the wound closure rate of HeLa/DDP and SiHa/DDP cells (Figures 7E–I). Similarly, the miR-92a-3p-mediated reduced invasion and migration of HeLa/DDP and SiHa/DDP cells were reversed by cotransfection with KLF4. EMT related protein expression levels were also examined using western blot. It was observed that miR-92a-3p knockdown resulted in a significant increase in E-cadherin expression, and a marked decrease in Vimentin and N-cadherin expression, whereas these effects of miR-92a-3p were abolished when KLF4 was knocked down in HeLa/DDP and SiHa/DDP cells (Figures 7J,K). Collectively, these results indicated that miR-92a-3p improved sensitivity of DDP-resistant CC cells to DDP *via* regulating KLF4 expression.

### High miR-92a-3p Expression Is Associated With Poor Prognosis of CC Patients Receiving DDP Therapy

**FIGURE 8:**
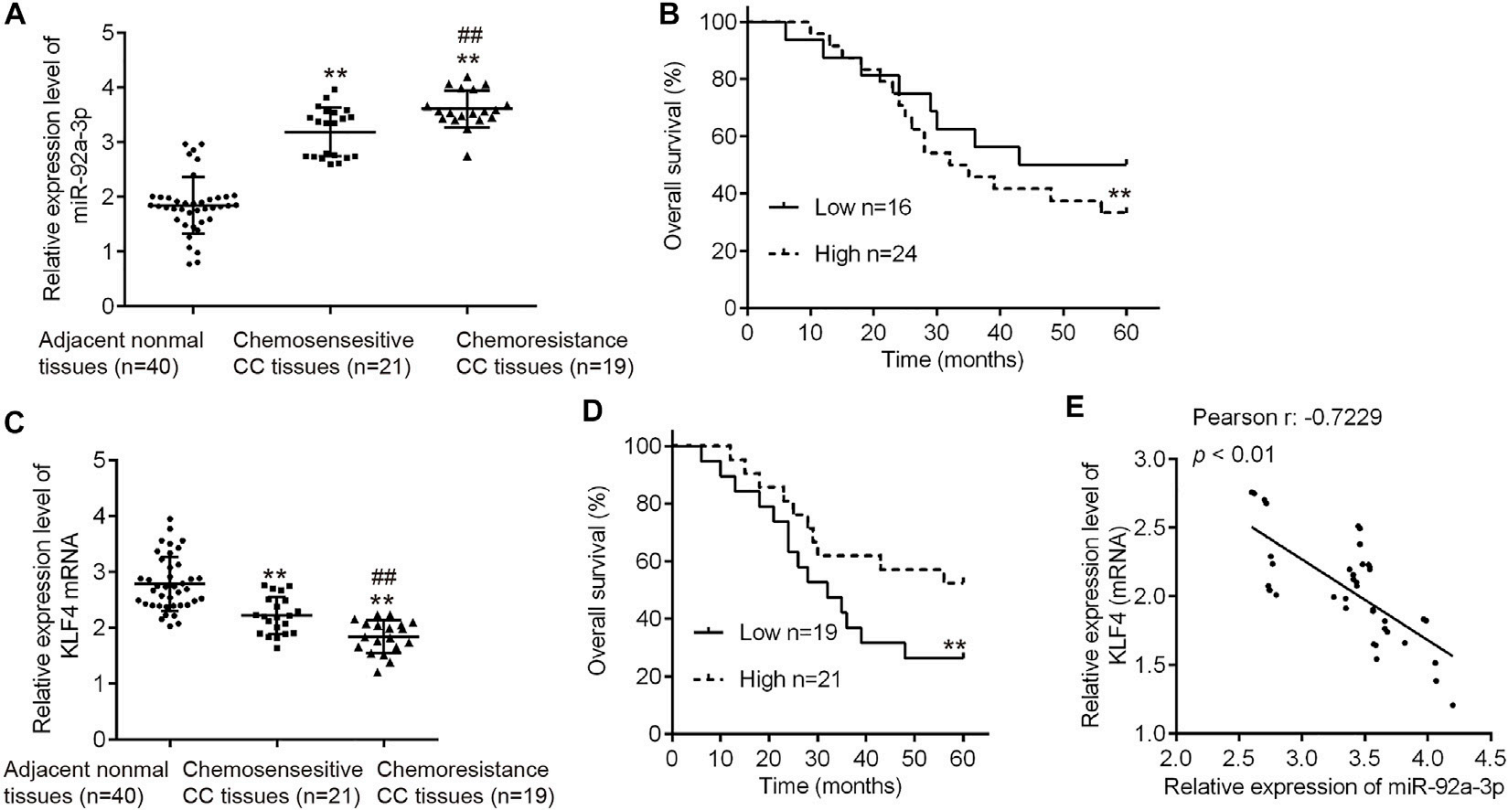
High miR-92a-3p expression is associated with poor prognosis of cervical cancer patients receiving DDP therapy. **(A)** miR-92a-3p levels in 40 patients (19 DDP-sensitive and 21 DDP-resistant cervical cancer tissues) were analyzed using qRT-PCR. **p < 0.01. *vs*. Adjacent normal tissues, ##*p* < 0.01. *vs*. Chemosensitive CC tissues. **(B)** Cervical cancer patients were separated into groups based on low or high miR-92a-3p expression. Kaplan-Meier survival curves were used to compare the overall survival rate between the two groups. **p < 0.01. **(C)** KLF4 levels in 40 patients who responded to DDP (n = 19) and who did not respond (*n* = 21) to DDP were analyzed using qRT-PCR. **p < 0.01. vs. Adjacent normal tissues, ##*p* < 0.01. *vs*. Chemosensitive CC tissues. **(D)** Cervical cancer patients were separated into groups based on low or high KLF4 expression. Kaplan-Meier survival curves were used to compare the overall survival rate between the two groups. **p < 0.01. **(E)** The correlation between miR-92a-3p and KLF4 was analyzed in cervical cancer tissues. *p* < 0.01.

Since miR-92a-3p was substantially upregulated in both HeLa/DDP and SiHa/DDP cells compared to parental cells, we thought it was important to find the clinical role in human CC. Therefore, we analyzed miR-92a-3p tissue levels in 40 patients who underwent DDP treatment (19 DDP-sensitive and 21 DDP-resistant CC tissues) using qRT-PCR. Compared with adjacent normal tissues, miR-92a-3p expression levels were significantly increased in DDP-sensitive and DDP-resistant CC tissues, and even higher in DDP-resistant CC tissues (Figure 8A). Kaplan-Meier analysis showed that patients exhibiting a high level of miR-92a-3p were correlated with shorter overall survival time (Figure 8B). In addition, we also analyzed the clinical relevance of KLF4 in human CC. As expected, compared with adjacent normal tissues, KLF4 expression levels were significantly decreased in DDP-sensitive and DDP-resistant CC tissues, and even lower in DDP-resistant CC tissues (Figure 8C). Next, CC patients (*n* = 21) with a low expression of KLF4 presented worse overall survival than the patients (*n* = 19) with a high expression of KLF4 (Figure 8D). In addition, in CC tissues, the expression of KLF4 was significantly negative correlated with that of miR-92a-3p (Figure 8E). All these data suggest that miR-92a-3p/KLF4 axis can regulate the clinical progression of CC.

## Discussion

In the present study, we found that miR-92a-3p was upregulated in cisplatin (DDP)-resistant CC cells. Knockdown of miR-92a-3p re-sensitized DDP-resistant cells to DDP, whereas overexpression of miR-92a-3p promoted DDP resistance in parental cells. Moreover, miR-92a-3p inhibition increased the sensitivity of DDP-resistant CC cells to DDP by directly targeting KLF4. Clinically, high miR-92a-3p expression was associated with shorter survival time in CC patients receiving DDP therapy.

Numerous studies in recent years have indicated that aberrant miRNA expression contributes to chemoresistance in various human malignancies, including CC (Zhang et al., 2018c; Yu M. et al., 2019). A previous study showed that miR-497 was significantly reduced in chemotherapy-resistant HeLa/DDP cells and contributed to DDP chemo-sensitivity by targeting transketolase (Yang et al., 2016). In another study, overexpression of miR-181a could inhibit the expression of osteopontin (OPN), induce cell apoptosis, restrain cell proliferation, and reduce DDP resistance in CC cells (Xu et al., 2019). In the present study, we screened potential miRNAs that may be crucial for DDP resistance in CC. *Via* retrieving the microarray data in the GEO dataset (GSE19611), 58 miRNAs were identified to have significant difference in expression between CC tissues and adjacent normal tissues, including several miRNAs that have been previously reported to be associated with the progression of CC. For example, it has been reported that miR-92a-3p, miR-221-3p, miR-196a, miR-146a and miR-21 promoted CC cell migration and invasion *in vitro* (Hou et al., 2014; Wei et al., 2017; Zhang et al., 2018d; Hu et al., 2018; Li et al., 2018), whereas miR-206, miR-383 and miR-497 inhibited cell proliferation and invasion in CC (Tao et al., 2018; Wang and Tian, 2018; Hu et al., 2019). In this study, we verified that miR-92a-3p was the most significantly upregulated microRNA in DDP-resistant cells compared to sensitive parental cells. Noteworthy, miR-92a has been previously reported to enhance DDP sensitivity of osteosarcoma (OS) cells (Liu et al., 2018). Therefore, we chose miR-92a-3p for further study.

Several studies have described the functions of miR-92a-3p in a variety of human malignancies, including CC (Mao et al., 2020; Jinghua et al., 2021; Wang et al., 2021). For example, miR-92a-3p regulated breast cancer cell proliferation and metastasis *via* regulating B-cell translocation gene 2 (BTG2) (Jinghua et al., 2021). Mao et al. found that the expression levels of miR-92a-3p were increased in gastric cancer tissues, and miR-92a-3p facilitated gastric cancer cell proliferation, DNA synthesis and cell invasion (Mao et al., 2020). In CC, miR-92a-3p was found to be upregulated in CC and promoted cell proliferation and invasion by targeting FBXW7 (Zhou et al., 2015). Luo et al. also demonstrated that miR-92a-3p promoted cell viability and invasion in CC *via* directly targeting Dickkopf-related protein 3 (DKK3) (Luo et al., 2017). However, little was known about the functions of miR-92a-3p in chemoresistance. In this study, we first established cisplatin-resistant HeLa/DDP and SiHa/DDP cell lines through treating with increased concentrations of DDP in a stepwise manner during each passage. Then, we tested miR-92a expression in HeLa/DDP and SiHa/DDP cell lines and miR-92a expression levels were significantly upregulated. Moreover, using gain- and loss-of-function approaches *in vitro*, we found that the inhibition of miR-92a-3p re-sensitized DDP-resistant CC cells to DDP, while overexpression of miR-92a-3p induced DDP resistance in sensitive parental cells, suggesting that the critical role of miR-92a-3p in regulating DDP resistance in CC.

It is well-known that miRNA regulates target gene expression by mediating target gene cleavage or inhibition of protein synthesis (Lin and Gregory, 2015). In the present study, using bioinformatics methods, KLF4 was predicted as the target gene of miR-92a-3p, which subsequently confirmed by a luciferase activity assay and western blot assay in CC cells. KLF4 is a member of the KLF-like factor subfamily of zinc finger proteins, which was recently reported to have a tumor suppressive role in a number of human cancers including bladder cancer, gastric cancer and pancreatic cancer (Ohnishi et al., 2003; Wei et al., 2005; Wei et al., 2008; He et al., 2015). Moreover, KLF4 has been demonstrated to be associated with chemotherapeutic resistance. For example, Yadav et al. found that KLF4 sensitized the colon cancer cell HCT-15 to cisplatin by altering the expression of HMGB1 and human telomerase reverse transcriptase (hTERT) (Yadav et al., 2019). Zhang et al. Showed that KLF4 could promote cisplatin-induced apoptosis by upregulating BIK expression in prostate cancer (Zhang L. et al., 2018). Chen et al. reported that KLF4 enhanced the sensitivity of cisplatin to ESCC cells through apoptosis induction and cell cycle arrest (Chen et al., 2017). Liu et al. demonstrated that KLF4 was significantly downregulated in cisplatin-resistant A549 cells and forced KLF4 expression inhibited cell growth and induced apoptosis, as well as suppressed the EMT process in cisplatin-resistant A549 cells (Liu et al., 2016). In our study, we demonstrated that inhibition of miR-92a-3p re-sensitized DDP-resistant CC cells to DDP, while overexpression of miR-92a-3p made CC parental cells resistant to cisplatin. To confirm whether miR-92a-3p exerts these effects through the regulation of KLF4, a rescue experiment was performed. As expected, KLF4 knockdown reversed miR-92a-3p inhibitor-induced cisplatin sensitivity in DDP-resistant cells. To conclude, these findings suggested that miR-92a-3p could enhance DDP resistance by regulating KLF4 expression.

Previous studies have reported that miR-92a-3p may be used as a prognostic biomarker to improve diagnostic efficiency in various types of cancers. For example, Lu et al. showed that the expression level of serum miR-92a-3p in gastric cancer patients was significantly downregulated, and the level was closely correlated with lymph node metastasis and tumor node metastasis stage of gastric cancer patients (Lu et al., 2021). Ren et al. showed that miR-92a-3p could be an independent prognostic factor in gastric cancer, and patients with high expression of miR-92a-3p experienced shorter survival (Ren et al., 2016). Meanwhile, higher expression levels of circulating exosomal miR-92a-3p were found to be significantly associated with pathologic stages and grades of the colorectal cancer patients (Fu et al., 2018). Our present study showed that miR-92a-3p was increased in CC tissues when compared with non-tumor tissues, and even higher in DDP-resistant CC tissues, while an opposite result was observed in the expression of KLF4. Meanwhile, high miR-92a-3p expression was correlated with short overall survival in CC patients who received DDP-based chemotherapy, supporting the potential application of miR-92a-3p to predict the prognosis of DDP-based chemotherapy. More importantly, a negative correlation was found between KLF4 and miR-92a-3p expression, which strongly supports the regulatory role of miR-92a-3p/KLF4 axis in DDP resistance in CC.

In conclusion, our data provides evidence that miR-92a-3p enhanced DDP resistance by regulating KLF4-mediated cell apoptosis and EMT in CC. Moreover, we verified the prognostic influence of miR-92a-3p and showed that high miR-92a-3p expression was associated with poor survival in patients receiving DDP therapy. Our findings suggest the miR-92a-3p might be a potential target for the prediction and treatment of CC patients with DDP resistance.

## Data Availability

The original contributions presented in the study are included in the article/Supplementary Material, further inquiries can be directed to the corresponding author.
