# Supplementary figures and images for: MicroRNA-92a-3p Enhances Cisplatin Resistance by Regulating Krüppel-Like Factor 4-Mediated Cell Apoptosis and Epithelial-to-Mesenchymal Transition in Cervical Cancer
Source: Front Pharmacol. 2022 Jan 14;12:783213. doi: 10.3389/fphar.2021.783213 (PMC8795743; doi:10.3389/fphar.2021.783213)

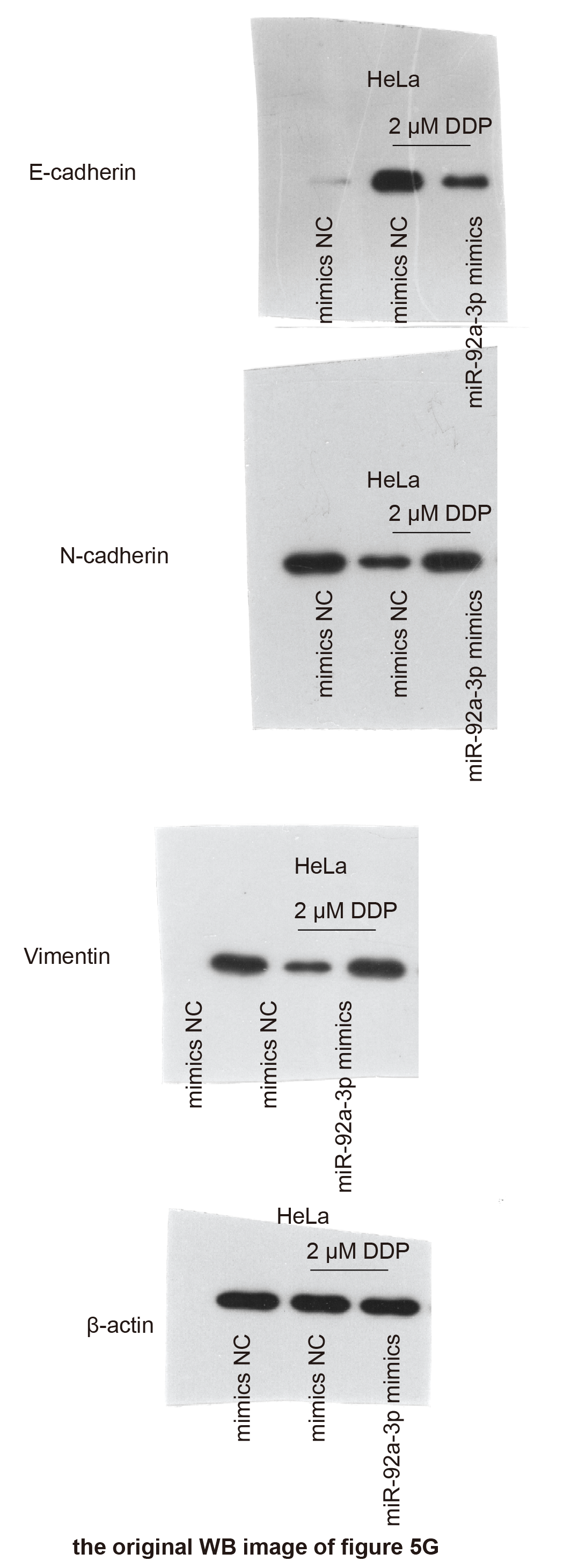

Supplement: Supplementary file 1 [file Image3.TIF]

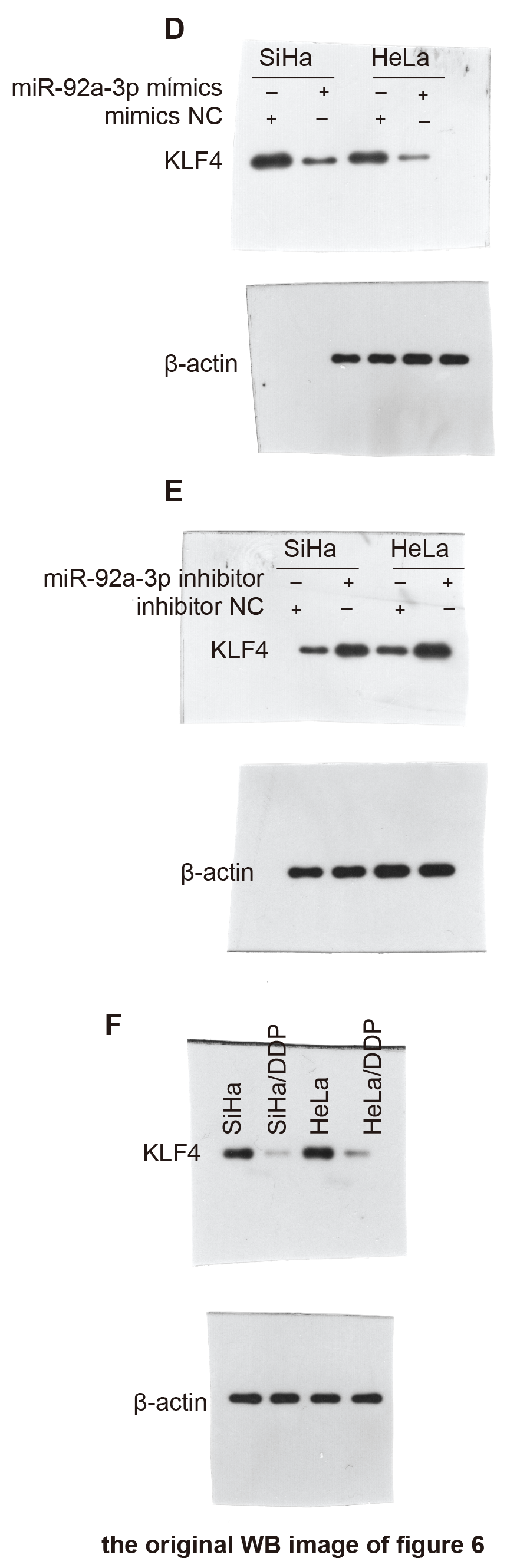

Supplement: Supplementary file 2 [file Image4.TIF]

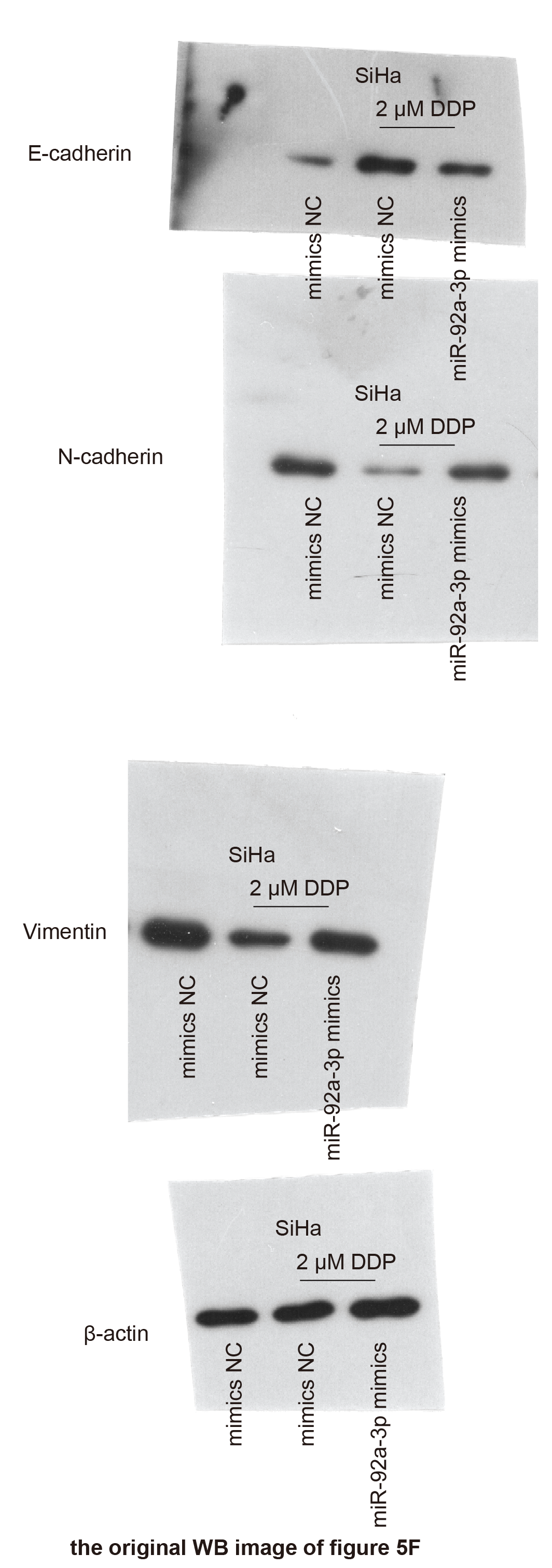

Supplement: Supplementary file 3 [file Image2.TIF]

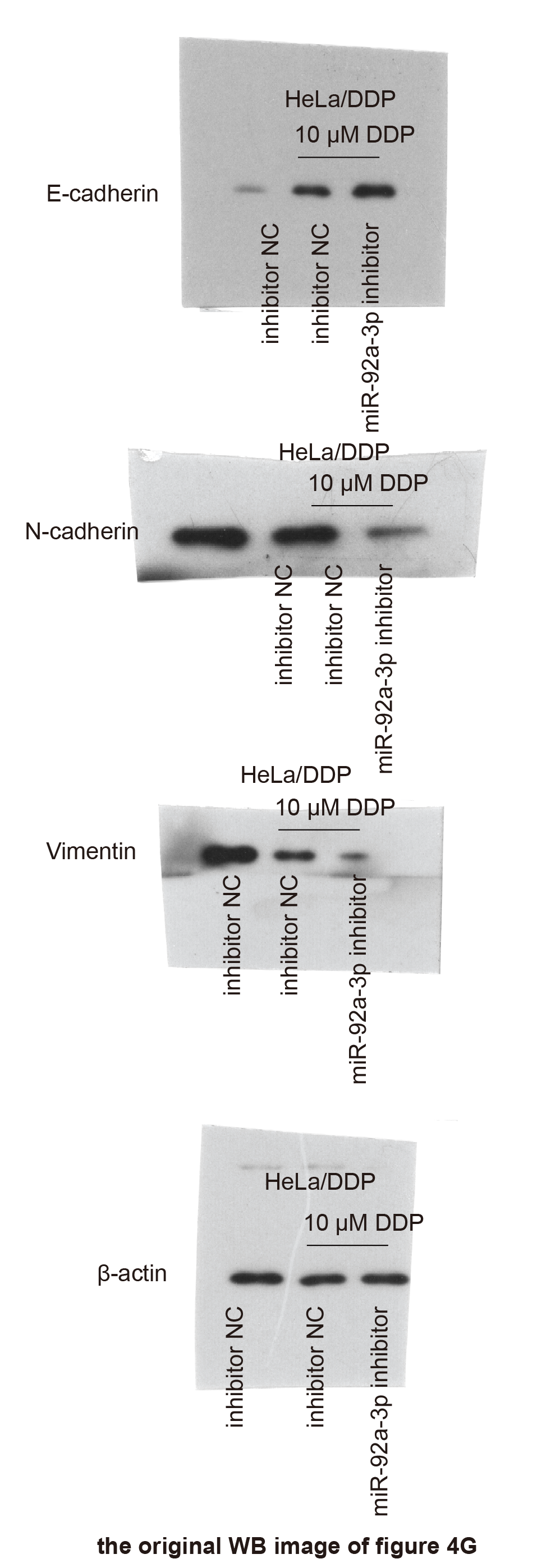

Supplement: Supplementary file 4 [file Image1.TIF]

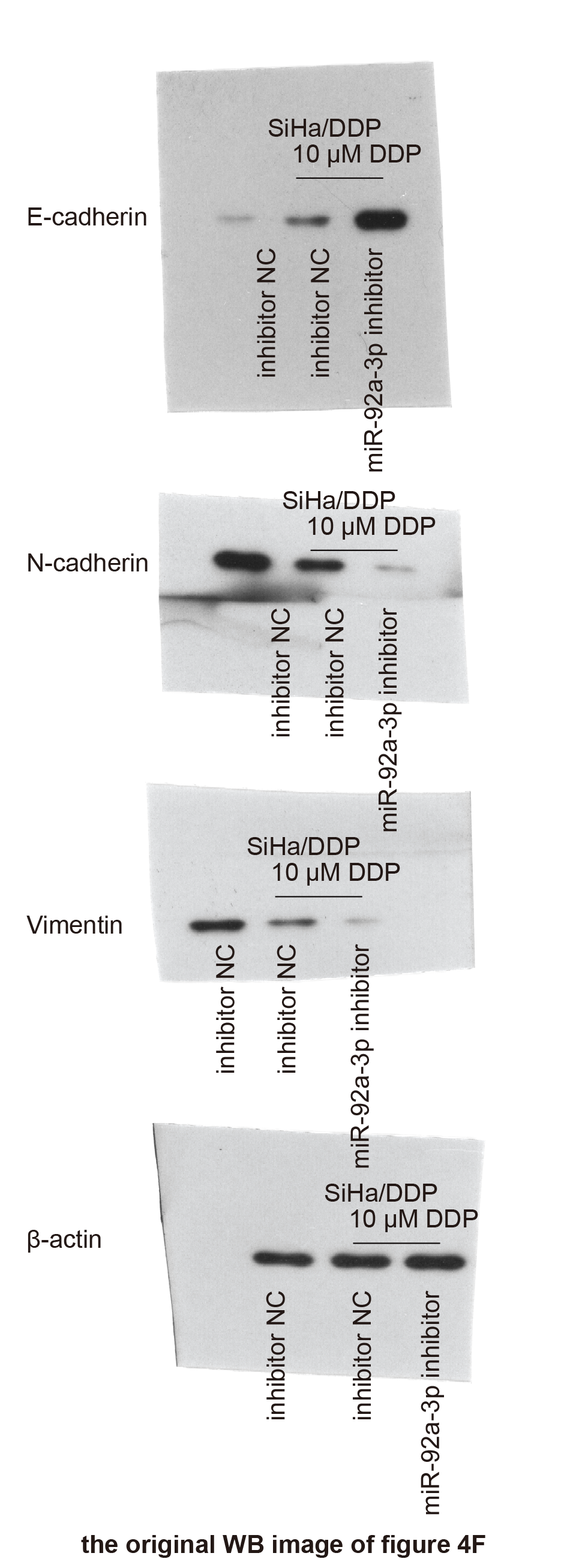

Supplement: Supplementary file 5 [file Image5.TIF]
